# Editing mitochondrial and chloroplast genomes: Genome editing technologies for plant organelles provide new opportunities in functional genomics and breeding

**DOI:** 10.1038/s44319-025-00675-3

**Published:** 2025-12-19

**Authors:** Joachim Forner, Ralph Bock

**Affiliations:** Max-Planck-Institut für Molekulare Pflanzenphysiologie, Am Mühlenberg 1, Potsdam-Golm, D-14476 Germany

**Keywords:** Methods & Resources, Organelles, Plant Biology

## Abstract

The article discusses recent progress with developing tools for organellar genome editing, highlights current and potential future applications, and analyzes benefits and limitations of genome editing technologies compared to organellar genome transformation.

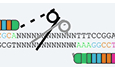

The engulfment and subsequent domestication of bacteria have equipped eukaryotic cells with two types of DNA-containing organelles: mitochondria—present in nearly all eukaryotes—and chloroplasts, also called plastids, that are present in plants, algae, and a few unicellular parasites. Both organelles play prominent roles in cellular energy metabolism by housing respiration (mitochondria) and photosynthesis (chloroplasts), the processes that sustain all eukaryotic life on the planet. During evolution, the genomes of mitochondria and plastids became greatly reduced by gene loss and large-scale transfer of genes from the organellar genomes to the nuclear genome. As a result, present-day organellar genomes typically contain less than 5% of the genes that their bacterial ancestors once had.

Both basic and applied research have a significant interest in technologies to genetically engineer organellar genomes. For example, a number of hereditary diseases in humans are caused by mutations in the mitochondrial DNA, which potentially could be repaired by genetic interventions. In plants, pollen sterility—one of the most important breeding tools also known as cytoplasmic male sterility—is conferred by mutations in the mitochondrial genome. Chloroplasts have emerged as superb low-cost production platforms for recombinant proteins and valuable metabolites (“green chemicals”), an area referred to as molecular farming (Bock, 2015). However, the development of engineering methods for organellar genomes has turned out to be a stony road, and despite enormous efforts, most organisms remain recalcitrant to the introduction of foreign DNA into their organelles. For example, stable transformation of the mitochondrial genome is currently possible only in baker’s yeast and the unicellular green alga *Chlamydomonas*, but not in any multicellular organism. Similarly, workable protocols for plastid transformation are available only for a handful of plant species.

## Organelle genome editing

Recently, genome-editing technologies have been adapted for organellar genomes (Kazama et al, 2019; Nakazato et al, 2021; Forner et al, 2022; Tan et al, 2022). They are technically less demanding than organellar transformation because they can be executed via nuclear transformation, which is routinely available in many species. For nuclear genome-editing, CRISPR/Cas-based systems have quickly become the dominant tool, with ever-expanding functionalities. They are easy to engineer, versatile, and predestined for multiplexing.

“[Genome-editing technologies] are technically less demanding than organellar transformation, because they can be executed via nuclear transformation.”

For organellar genome editing, however, CRISPR/Cas-based methods are unsuitable. This is because they consist of two essential components: a protein that catalyzes the genomic modification, and an RNA molecule, the so-called single guide RNA (sgRNA), that provides the target specificity. While it is straightforward to direct nucleus-encoded proteins to plastids or mitochondria by adding an appropriate N-terminal targeting sequence—the so-called transit peptide—this is, unfortunately, not possible for foreign RNA molecules; there is currently no way to deliver sgRNAs to plastids or mitochondria.

## TALE editing technologies

**Figure 1:**
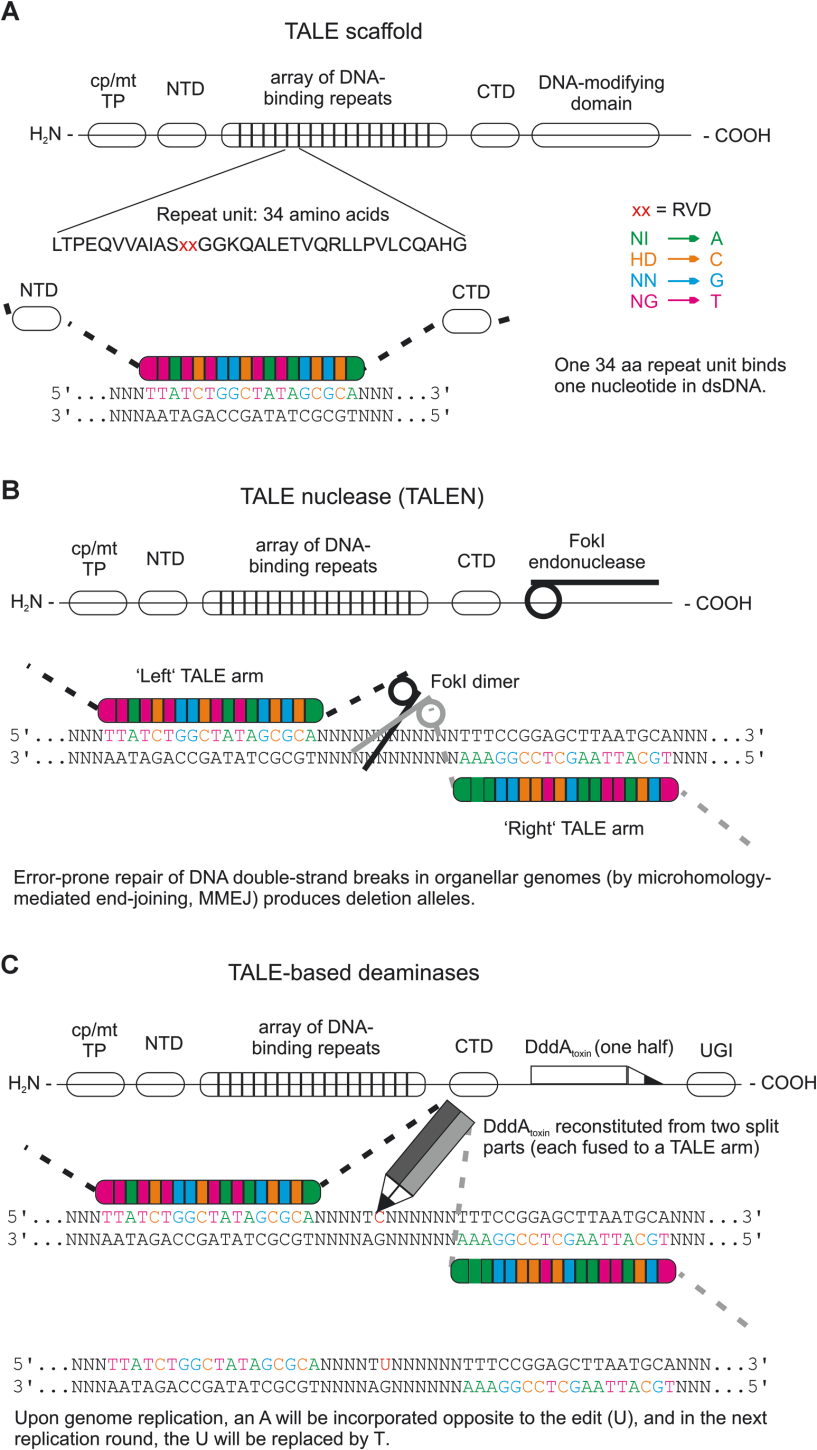
DNA modifications in organellar genomes by protein-based genome editing tools. (**A**) Basal architecture of the TALE (transcription activator-like effector) scaffold. H_2_N- N-terminus, cp/mt TP plastid or mitochondrial transit peptide for protein targeting to the organelle, NTD N-terminal domain, CTD C-terminal domain, -COOH C-terminus, RVD repeat variable di-residue. One repeat unit of 34 amino acids in the DNA-binding array binds exactly one nucleotide in the target DNA (with the 5’-to-3’ direction in the DNA corresponding to the N-to-C-terminal direction in the protein). Nucleotide-specificity is conferred by the identity of the two amino acids (RVD) at positions 12 and 13 in the individual repeat unit. This TALE code is depicted in the right. Amino acids are given in the one-letter code. (**B**) Architecture of TALE nucleases (TALENs). Fusing the FokI endonuclease domain (depicted as half scissors) to the C-terminus of the TALE scaffold creates a target-specific endonuclease acting on double-stranded DNA. Since FokI only works as a dimer, two TALE arms each providing a FokI monomer are needed to form a functional endonuclease (depicted as complete scissors). The DNA cut occurs between the two binding sequences. Deletions in organellar genomes can be generated when the break in the DNA is not repaired by homologous recombination, which would restore the wild-type sequence, but instead, is fixed by microhomology-mediated end-joining (MMEJ). The homologous template for MMEJ can come from anywhere in the genome, and the sequence utilized is largely unpredictable. (**C**) TALE-based deaminases acting as base editors. DddA_toxin_: catalytically active domain of the *Burkholderia cenocepacia* double-stranded DNA deaminase toxin A; UGI: uracil-DNA glycosylase inhibitor. DddA_toxin_ deaminates cytidines in double-stranded DNA in a non-sequence-specific manner. To convert DddA into a sequence-specific editing tool, it is split into two parts that have no catalytic activity on their own (depicted as half pencils). When the two halves are fused to suitable TALE repeat arrays, they can be directed to a specific sequence in the DNA, reconstitute the active enzyme, and site-specific cytidine deamination can occur. Note that the basic design is analogous to the TALEN design, with the exception that the two TALE arms are fused to two different polypeptide moieties (the N-terminal or the C-terminal part of DddA_toxin_). Figure redrawn and modified from (Tan et al, 2022).

Consequently, organellar DNA editing requires protein-only genome-editing reagents (Table 1; Fig. 1). For this reason, currently available genome-editing methods for organelles largely rely on so-called TALEs (transcription activator-like effectors), DNA-binding proteins secreted by some plant pathogenic bacteria into infected host cells to manipulate gene expression to their benefit. TALEs are composed of repetitive units of 34 amino acids, with each repeat unit binding a single base pair in the DNA. Nucleotide specificity is mediated by two variable amino acid residues at positions 12 and 13 within each repeat unit (repeat variable di-residue or RVD; Fig. 1A). The combination of histidine in position 12 (H12) and aspartate in position 13 (D13), for example, will cause the respective unit to bind to a cytosine (C) residue in the DNA. This binding behavior results in a simple one-to-one mapping of repeat units to nucleotides, with the N-to-C-terminal direction of the repeat arrays in the TALE protein matching the 5’-to-3’ direction of the nucleotides in the target DNA. The different repeat units can be combined in a modular fashion, so that any given DNA sequence can be targeted according to the TALE code as defined by the RVDs (Fig. 1A). The high number of repeats involved in DNA binding ensures that the target site is unique in the genome and that off-target effects are relatively rare.

**Table 1:** Comparison of genome editing and organelle transformation technologies for site-specific modification of plastid and mitochondrial genomes.

	Genome editing	Genetic transformation
Modification principle	DNA cut, nucleoside deamination	Homologous recombination
Modifying agent	Protein effectors based on TALE repeats (integrated as transgenes into the nucleus)	DNA with sequence homology to the organellar genome
Scope of possible changes	Insertions: no Deletions: yes, but size is neither predictable nor controllable Base changes: yes, but only certain types Transgenesis: no	Insertions: yes Deletions: yes Base changes: yes (any) Transgenesis: yes
Precision	Medium to high	Very high
Off-targets within the compartment	Present (at varying frequencies); base editors: bystander mutations and off-target editing; TALENs: genome rearrangements	Largely absent (rarely: genome rearrangements)
Off-targets in other compartments	Present	Largely absent (rarely: co-transformation of the nuclear genome can occur)
Accessibility of sites in the genome	Limited (e.g., preference for TC by cytosine base editor)	Unrestricted
Selection for the intended DNA change	Usually not possible, genotyping necessary	Possible (via linkage to a nearby selectable marker gene)
Species range	All species whose nuclear genome can be transformed	Only a small number of species
Technical demand	Low to medium	Medium to high (gene gun, tissue culture)
Multiplexing	Limited	Limited
Auxiliary transgene requirements and post-transformation removal options	Usually necessary (in the nucleus), can be removed by outcrossing or site-specific recombination	Necessary (selectable marker gene), can be removed by repeat-mediated homologous recombination or site-specific recombination
Regulatory status of modified plants	Deregulated in many countries	Regulated as GMOs

“… organellar DNA editing requires protein-only genome-editing reagents.”

The original TALEs expressed by pathogenic bacteria are directed to the nucleus of the host cell via a suitable localization signal. In the host nucleus, they increase the expression of specific target genes by a transcription activation domain. For application in organellar genome editing, the nuclear localization signal and the transcription activation domain are removed and replaced by an N-terminal transit peptide for import into either plastids or mitochondria and a DNA-modifying domain at the C-terminus (Fig. 1). If the DNA-modifying domain is an endonuclease such as the restriction endonuclease FokI, it creates a TALE nuclease (TALEN) (Fig. 1B). As FokI only works as a dimer, two TALEN arms are required to cut the target DNA. The two proteins must bind the DNA at the same time, facing each other in tail-to-tail orientation. The endonucleolytic cut occurs in the spacer region between the two binding sites (Fig. 1B). As repair of the resulting DNA double-strand breaks is prone to error, this regularly generates mutations such as small deletions in nuclear genomes.

In contrast to DNA-repair in the nucleus, double-strand breaks in organellar genomes are usually repaired by homologous recombination (Kohl and Bock, 2009), which is much less error-prone, and does not readily generate deletions, unless repair utilizes microhomologies elsewhere in the genome (Forner et al, 2023). To address this limitation, site-specific base editors have been developed where a nucleoside deaminase domain is fused to the array of DNA-binding repeats (Fig. 1C).

## Site-specific mutagenesis

A key discovery has been the identification of a cytidine deaminase that acts on double-stranded DNA. The enzyme, DddA, was isolated from the bacterium *Burkholderia cenocepacia* (Mok et al, 2020), which uses it to kill competing bacteria. The deaminase domain of DddA, referred to as DddA_toxin_, would non-specifically deaminate cytidines all over the genome. To avoid these genotoxic effects, the domain is split into an N-terminal and a C-terminal part, each of which alone is not catalytically active. When brought together and directed to a specific site in the genome, the two parts reconstitute and deaminate cytidines within reach.

In organellar base editors, this is accomplished by fusing each of the two parts of the deaminase domain to a TALE arm, thereby generating a TALE-based DNA deaminase (DddA-derived cytosine base editor, DdCBE; Fig. 1C). Similar to the TALEN cut, the base modification occurs in the spacer region between the two TALE binding sites (Fig. 1B,C). The reaction product of cytosine deamination is not thymine, but uracil, which is efficiently removed by base excision repair by the enzyme uracil-DNA glycosylase. Since uracil-DNA glycosylase action would prevent the edit from becoming fixed in the genome, a uracil-DNA glycosylase inhibitor domain (UGI) is additionally fused to the base editor (Fig. 1C). To fix the desired base change, DNA replication is necessary. In the first replication round, an adenosine is incorporated in the opposite strand through complementary base pairing with uracil, which then causes incorporation of a thymine in the second replication round, thus fixing the edited allele. In general, cytosines at several positions in the spacer can be edited, without any pronounced strand preference. Such C-to-T changes in the neighborhood of the actual target nucleotide are referred to as bystander mutations.

Currently, there are no known adenosine deaminases that would act on double-stranded DNA, but in combination with DddA, adenosine deaminases that naturally act on single-stranded RNA can be used to induce A-to-G base editing in organelles. A mutated version of DddA_toxin_ (E1347A) cannot deaminate cytosine anymore, but still binds double-stranded DNA and melts it locally, so that single-stranded DNA becomes available to an adenosine deaminase domain fused to the editor. Thus, TALE-based cytosine deaminases can be converted to adenosine deaminases by introducing the E1347A mutation into DddA_toxin_ and replacing the UGI with an adenosine deaminase domain that acts on single-stranded nucleic acids. Adenosine is oxidatively deaminated to inosine, which base-pairs like guanosine, so, again, replication is needed to fix the edit. Using the unmutated form of DddA_toxin_ in this setting will create a dual editor that catalyzes both C-to-T and A-to-G mutations in the target region (Mok et al, 2022; Zhou et al, 2024).

Up to now, targeted genome modification in plant organelles has mainly been used to inactivate genes by inducing deletions with TALENs, or nonsense or missense mutations with TALE-based deaminases (Fig. 1; Maliga, 2022; Tan et al, 2022; Arimura and Nakazato, 2024). These approaches have enabled the analysis of organellar gene functions, which has been particularly useful in assessing mitochondrial open reading frames as candidate genes for cytoplasmic male sterility (Kazama et al, 2019; Takatsuka et al, 2022).

“Up to now, targeted genome modification in plant organelles has mainly been used to inactivate genes…”

In chloroplast genomes, a few point mutations that confer resistance to antibiotics or herbicides have been introduced by base editing (Nakazato et al, 2025), but the number of proteins that are encoded in the plastid genome and represent targets of commercially relevant herbicides is very limited. It is, therefore, currently not obvious how genome editing in plant organelles could lead to novel applications in breeding and agricultural biotechnology. It is conceivable that polymorphisms in plastid and/or mitochondrial genes that play a role in environmental adaptation could be introduced by genome editing into crops to produce elite varieties that are adapted to local environmental conditions. Unfortunately, our knowledge about the role of organellar genomes—and specific alleles—in environmental adaptation is still so poor that such targeted approaches currently cannot yet be pursued. However, organellar genomes do have a strong impact on plant phenotypes and crop performance in the field (Flood et al, 2020), suggesting that there is significant potential in producing improved locally adapted crop varieties by editing plastid and mitochondrial genomes.

“…organellar genomes do have a strong impact on plant phenotypes and crop performance […], suggesting that there is significant potential in producing improved locally adapted crop varieties by editing plastid and mitochondrial genomes.”

**Figure 2:**
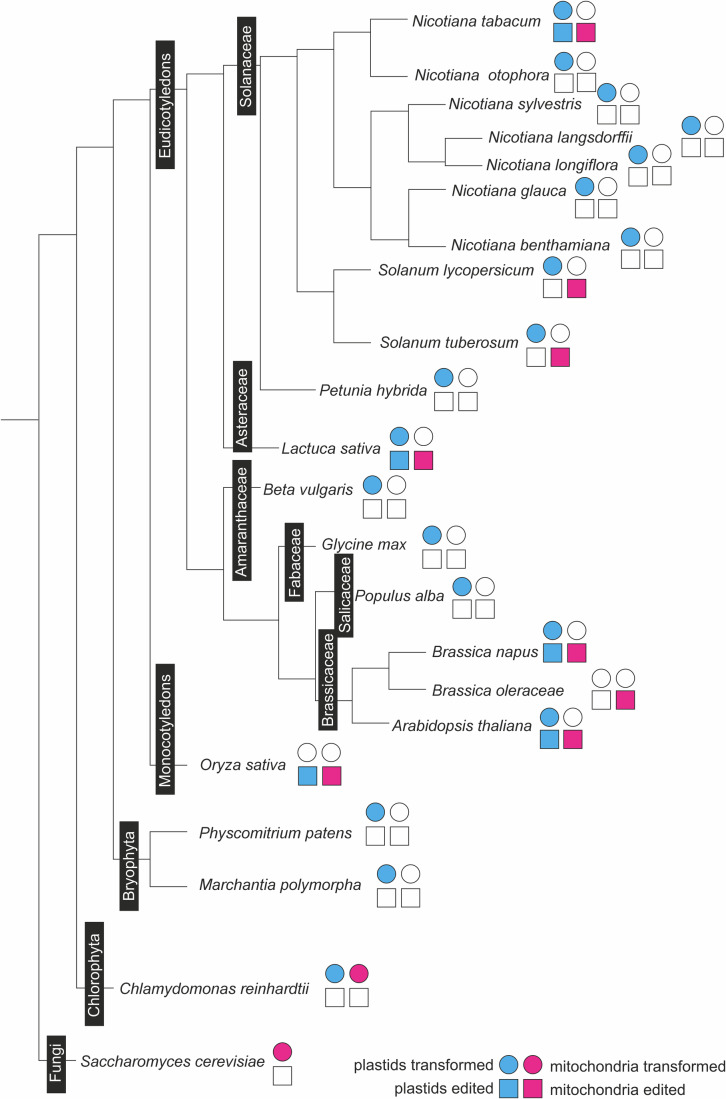
Overview of the current species range of organelle transformation and organelle base editing in plants. Shown is a simplified phylogenetic tree, including the unicellular green alga *Chlamydomonas reinhardtii* and the yeast *Saccharomyces cerevisiae*, the two only species in which mitochondrial transformation is currently possible. Species in which organelle transformation has been reproducibly accomplished are marked with colored circles (cyan: plastid transformation; magenta: mitochondrial transformation). Selected species in which organellar genome editing has been achieved are marked with a colored square (cyan: plastids; magenta: mitochondria). In most taxonomic groups, organelle transformation is not yet possible, including very large groups such as the gymnosperms and the monocotyledonous plants with the world’s most important staple crops rice, wheat, and maize. Of the more than 300 families of dicotyledonous plants, less than ten families have species, in which plastids are transformable. The relatively high number of transformable species in the Solanaceae (nightshade) family is due to the generally favorable tissue culture and regeneration properties of many species. Note that branch lengths of the tree are uniform and do not correspond to phylogenetic distance.

The major attraction of genome editing lies in its relatively low technical requirements. While genetic transformation technologies for organellar genomes are technically demanding and involve laborious and time-consuming procedures, genome editing can be executed relatively easily and, in principle, should work in all organisms whose nuclear genome is transformable (Table 1; Fig. 2). For example, in the absence of mitochondrial transformation technology for any plant species, the development of editing methods for mitochondria (Fig. 1) has made the mitochondrial genome of seed plants amenable to genetic changes (Fig. 2; Tan et al, 2022). Likewise, large taxonomic groups for which chloroplast transformation is unavailable, such as the monocotyledonous seed plants, have become accessible to plastid genome editing (Fig. 2).

## Benefits and limits

However, compared to organelle transformation, organellar genome editing also has a number of limitations. The most serious ones are its restriction to certain mutation types that can be induced in the target genome; and the impossibility to introduce foreign DNA sequences (transgenes) into organellar genomes. The latter is a particularly serious shortcoming in that it severely limits the range of applications that can be pursued. For example, while mitochondrial candidate genes for cytoplasmic male sterility can be confirmed by knock-out analysis using genome editing which restores pollen fertility, the introduction of cytoplasmic male sterility into new crops will require the transfer of such genes into the mitochondrial genome by transformation. Also, the precision of organelle transformation methods is much higher than that of genome-editing methods, given that organelle transformation occurs by homologous recombination, which provides full control over the genome. For the same reason, off-target effects occur less frequently in organelle transformation (Table 1).

Thus, organelle transformation is the method of choice for all species in which it is available. Organellar genome editing provides a particularly useful tool for functional genomics in plant mitochondria, that is, the investigation of mitochondrial gene functions by producing knock-outs, where no transformation technology exists (Kazama et al, 2019; Takatsuka et al, 2022; Forner et al, 2023). Most genes encoded in the mitochondrial genome are likely essential for plant survival, but by using established tools for tissue-specific, developmental stage-specific and/or inducible expression of the nucleus-encoded genome editing reagents, it will likely be possible to construct conditional knock-out mutants of mitochondrial genes that provide new insights into their function during plant development.

“… organelle transformation is the method of choice for all species in which it is available.”

In chloroplasts, the application of genome editing tools will likely remain restricted to a few niche applications that cannot be conducted in transformable model species and/or address non-conserved features of the organellar genome in a particular (non-transformable) species. For example, the functional analysis of open reading frames that do not exist in transformable species can be pursued by genome editing. Also, structure-function relationships in organellar genes can be elucidated using mutagenesis based on genome editing (Forner et al, 2022). Especially in plant mitochondria, we still know very little about the control elements of gene expression, including promoters, 5’ and 3’ untranslated regions (UTRs), and their role in mRNA stability and translation, and the *cis*-elements that govern RNA processing, RNA editing and intron splicing. Base editing and site-directed mutagenesis techniques will likely prove useful in identifying consensus sequences for many of these steps in organellar gene expression. We also know next to nothing about the sequences controlling the replication of organellar genomes, and their impact on genome segregation, genome inheritance, and competition between different organellar genotypes when present in the same individual. By generating allelic diversity in the genomes and, in particular, in candidate sequences potentially involved in these processes (Sobanski et al, 2019), new fundamental insights could be obtained. Nonetheless, the greatly superior precision and the much broader application range of organelle transformation (Table 1) will likely make genome editing obsolete in all those species for which workable methods for organelle transformation become available.

## Potential applications and regulation

Similar to most applications of DNA editing in nuclear genomes, the editing of organellar genomes produces mutants rather than transgenic organisms. In crop plants, genome-edited new varieties are similar to, and in fact often indistinguishable from, varieties obtained by conventional mutation breeding for instance by X-ray or γ-ray irradiation, or chemical mutagenesis. A transgenic intermediate can be involved in the generation of the genome-edited variety, because stable expression of the genome-editing reagents is usually more efficient than their introduction by transient transfection methods. However, in all sexually propagated crops, the transgene(s) can be easily crossed out after editing has occurred, resulting in a transgene-free new variety.

Therefore, it is becoming increasingly accepted that genome editing should be seen as just another mutagenesis technique rather than a transgenic technology. In many countries, genome-edited crops are therefore considered equivalent to crops obtained by conventional mutagenesis techniques and, thus, are not regulated as transgenic organisms (GMOs). Among the few countries that still regulate genome-edited crops as GMOs are the member states of the European Union (Buchholzer and Frommer, 2023). This results in a high regulatory burden and effectively, in a ban of the technology. To address these regulatory and policy deficiencies, in 2023, the European Commission has adopted a proposal that would exempt genome-edited crop varieties from GMO legislation, if similar or identical genetic changes could have been obtained through conventional breeding. In 2024, the European Parliament voted in favor of the reform, but it must still be agreed upon by the EU member states. In the interest of promoting agricultural innovations for food security and climate change adaptation in Europe, it is hoped that a consensus can be reached soon.

“… it is becoming increasingly accepted that genome editing should be seen as just another mutagenesis technique rather than a transgenic technology.”

## Supplementary information

Peer Review File
