# Supplementary material for: Editing mitochondrial and chloroplast genomes: Genome editing technologies for plant organelles provide new opportunities in functional genomics and breeding
Source: EMBO Rep. 2025 Dec 19;27(2):269–75. doi: 10.1038/s44319-025-00675-3 (PMC12852748; doi:10.1038/s44319-025-00675-3)
Supplement: Supplementary file 1 — Peer Review File [file 44319_2025_675_MOESM1_ESM.pdf]

EMBO reports

Editing mitochondrial and chloroplast genomes

Joachim Forner and Ralph Bock

Corresponding author(s): Ralph Bock (rbock@mpimp-golm.mpg.de) , Joachim Forner (Forner@mpimp-golm.mpg.de)

Review Timeline:

Submission Date:	3rd Dec 25
Accepted:	4th Dec 25

Editor: Holger Breithaupt

Transaction Report: This article was editorially reviewed.
